# Counteracting Bacterial Motility: A Promising Strategy to Narrow *Listeria monocytogenes* Biofilm in Food Processing Industry

**DOI:** 10.3389/fmicb.2021.673484

**Published:** 2021-06-02

**Authors:** Ibtissem Doghri, Tamazight Cherifi, Coralie Goetz, François Malouin, Mario Jacques, Philippe Fravalo

**Affiliations:** ^1^Département de Pathologie et Microbiologie, Faculté de Médecine Vétérinaire, Université de Montréal, Saint-Hyacinthe, QC, Canada; ^2^Regroupement de Recherche pour un Lait de Qualité Optimale (Op+Lait), Montreal, QC, Cananda; ^3^Chaire de Recherche en Salubrité des Viandes (CRSV), Montreal, QC, Cananda; ^4^Département de Biologie, Faculté des Sciences, Université de Sherbrooke, Sherbrooke, QC, Canada

**Keywords:** *Listeria monocytogenes*, biofilm, motility, antibiofilm activity, food Industry

## Abstract

*Listeria monocytogenes* (*L. monocytogenes*) is often associated with processed food as it can form biofilms that represent a source of contamination at all stages of the manufacturing chain. The control and prevention of biofilms in food-processing plants are of utmost importance. This study explores the efficacy of prospect molecules for counteracting bacterial mechanisms leading to biofilm formation. The compounds included the phytomolecule tomatidine, zinc chloride (ZnCl_2_), ethylenediaminetetraacetic acid (EDTA), and a more complexed mixture of bacterial compounds from coagulase-negative staphylococci (CNS exoproducts). Significant inhibition of *L. monocytogenes* biofilm formation was evidenced using a microfluidic system and confocal microscopic analyses (*p* < 0.001). Active molecules were effective at an early stage of biofilm development (≥50% of inhibition) but failed to disperse mature biofilms of *L. monocytogenes*. According to our findings, prevention of surface attachment was associated with a disruption of bacterial motility. Indeed, agar cell motility assays demonstrated the effectiveness of these molecules. Overall, results highlighted the critical role of motility in biofilm formation and allow to consider flagellum-mediated motility as a promising molecular target in control strategies against *L. monocytogenes* in food processing environments.

## Introduction

Numerous epidemics of listeriosis caused by *Listeria monocytogenes (L. monocytogenes)*, a Gram-positive foodborne pathogen, have occurred in industrialized countries (Hamon et al., 2006; Sofos and Geornaras, 2010). The disease caused by this bacterium is acquired by ingesting contaminated food products and mainly affects susceptible individuals such as pregnant women, neonates, older adults, and immunocompromised individuals (Vazquez-Boland et al., 2001; Sofos and Geornaras, 2010). Listeriosis can manifest as gastroenteritis but may also manifest as severe listeriosis resulting in meningitis, encephalitis, mother-to-fetus infections and septicaemia, resulting in death in 25–30% of cases (Hamon et al., 2006).

*Listeria monocytogenes* is an ubiquitous bacterium mainly found in water, soil, and forage. For humans, the consumption of ready to eat (RTE) contaminated food products represents the main transmission mode (Allerberger and Wagner, 2010). The most sensitive food products are meat-based RTE, uncooked sea food and raw milk cheeses (Evans and Redmond, 2014; Kleta et al., 2017). *L. monocytogenes* is able to adapt and survive to the most constraining conditions such as low temperatures, UV radiation, low pH, and high osmolarity (Chaturongakul et al., 2008). It can form biofilms at all stages of the food-processing chain (Evans and Redmond, 2014; Cherifi et al., 2017). In fact, biofilms have been found on surfaces made of polystyrene, glass, and stainless steel (Cherifi et al., 2017; Reis-Teixeira et al., 2017). This ability may allow it to circumvent current hygienic practices, especially where less accessible niches remain, leading to persistent contamination of the manufactured food products (Tauxe, 2002; Chang et al., 2012).

*Listeria monocytogenes* has four to six peritrichous flagella per cell (Schirm et al., 2004). Flagellum-mediated motility is important for biofilm formation by several Gram-negative bacteria (O’Neil and Marquis, 2006) and in the case of *L. monocytogenes*, flagella are implicated as surface adhesins in early surface attachment (Vatanyoopaisarn et al., 2000).

There is a clear need to develop new strategies to control biofilm formation in food processing environments (Yin et al., 2019). One of the promising strategies is the discovery of new active molecules that target bacterial adhesive properties and biofilm formation without affecting bacterial viability in order to avoid the development of resistance following a life-threatening selective pressure. Their use may be then exclusive or combined in addition to antimicrobial treatments. To develop such a strategy, compounds of different origins were investigated in this study. First, molecules of microbial origin were investigated. For instance, cell-free supernatants of coagulase-negative staphylococci (CNS) were used. Our group previously demonstrated the ability of these CNS to inhibit biofilm formation of other staphylococci isolates producing large amounts of biofilm (Goetz et al., 2017). In addition, we evaluated the activity of tomatidine, a steroid alkaloid isolated from the tomato plant. Tomatidine has been described as an anti-virulence molecule against strains of *Staphylococcus aureus* (Mitchell et al., 2012) as well as having the ability to potentiate the effect of aminoglycoside antibiotics against the *Bacillales* order (*Listeria*, *Bacillus*, and *Staphylococcus* spp.) through inhibition of the bacterial ATP synthase and interference with energy production (Guay et al., 2018; Lamontagne Boulet et al., 2018). Such an effect was amplified against *S. aureus* small colony variants (SCVs), which are deficient respiratory forms that produce high amounts of biofilm (Mitchell et al., 2011, 2013). Besides, we also investigated the antibiofilm activity of zinc chloride since some studies reported antibiofilm activity against pathogens such as *Salmonella typhimurium*, *Escherichia coli*, *S. aureus* (Gu et al., 2012; Wu et al., 2013). Finally, EDTA was used as a reference antibiofilm molecule based on a study demonstrating that when used at low concentration, it displayed an antibiofilm activity against *L. monocytogenes* without affecting cell viability (Chang et al., 2012).

Therefore, the objective of this study was to test and characterize the ability of these compounds to counteract directly or indirectly biofilm formation by evaluating their effect on bacterial motility, adhesion to surfaces, biofilm formation and biofilm dispersion for various strains of *L. monocytogenes* isolated from food-processing environments.

## Materials and Methods

### Bacterial Strains and Growth Conditions

Four biofilm-forming strains of *L. monocytogenes* were used in the present study, LM 2A51, LM C97, LM 3C15, and LM 3C24. These strains were isolated from the floor of different areas in the pork slaughterhouses after sanitation procedures (Table 1) and belong to the serotypes 1/2a, 1/2a, 1/2a, and 1/2b, respectively, and they were characterized by Pulsed Field Gel Electrophoresis (PFGE) and are referred to the following PFGE types LS4, LS19, LS21, and LS16, respectively, in Cherifi et al. (2020). Five coagulase-negative staphylococci (CNS) of bovine origin were used to generate cell-free supernatants with an anti-biofilm activity (Goetz et al., 2017). These CNS isolates were obtained from the Mastitis Pathogen Culture Collection (MPCC) which is managed by the Mastitis network (Saint-Hyacinthe, QC, Canada; Dufour et al., 2019). Bacteria were preserved in brain heart infusion broth (BHI, Becton Dickinson and Company, United States) with the addition of 15% glycerol at −80°C. Prior to experiments, strains were thawed and plated on blood agar for *L. monocytogenes* (Oxoid, England) and BHI agar (BHIA) for CNS and were cultivated in aerobic conditions at 30 and 37°C, respectively.

**TABLE 1:** Bacterial strains and culture conditions.

**Strain**	**Origin**	**References and/or source**	**Culture conditions**
***Listeria monocytogenes***			
*Listeria monocytogenes* C97 (LM C97)	Floor-Refrigeration area (2013)	Cherifi et al., 2020 CRSV*	Blood agar medium 30°C 24 h
*Listeria monocytogenes* 3C15 (LM 3C15)	Floor-Lairage area (2014)	Cherifi et al., 2020 CRSV	
*Listeria monocytogenes* 3C24 (LM 3C24)	Floor-Lairage area (2014)	Cherifi et al., 2020 CRSV	
*Listeria monocytogenes* 2A51-1 (LM 2A51-1)	Floor-Lairage area (2013)	Cherifi et al., 2020 CRSV	
***Coagulase Negative Staphylococcus (CNS)***			
*Staphylococcus chromogenes* C	Bovine Mastitis	Goetz et al., 2017	BHIA 37°C 24 h
*Staphylococcus chromogenes* D	Bovine Mastitis	Goetz et al., 2017	
*Staphylococcus chromogenes* E	Bovine Mastitis	Goetz et al., 2017	
*Staphylococcus simulans* F	Bovine Mastitis	Goetz et al., 2017	
*Staphylococcus simulans* H	Bovine Mastitis	Goetz et al., 2017	

*CRSV: Research chair for meat safety – Vet Faculty- University of Montreal.

### Preparation of Active Molecules

#### Production of CNS Cell-Free Supernatants

The five CNS isolates (C, D, E, F, and H) were grown in BHI supplemented with 0.25% w/v glucose (BHIG) in 6-wells microtiter plates (Corning Costar #3516, United States) (Goetz et al., 2017). Colonies from BHIA plates were suspended in BHIG to a 0.5 McFarland standard and 9 mL were distributed in each well of the microtiter plate. The microtiter plates were then incubated for 24 h at 37°C. After the incubation, the supernatants (SN) were collected by centrifugation at 4,000 rpm for 20 min at 4°C and filter-sterilized through a 0.2 μm membrane then stored at −80°C until use.

#### Preparation of EDTA and Zinc Chloride Solutions

Filter sterilized ethylenediaminetetraacetic acid (EDTA, 10 mM, pH 8.0) (Sigma Aldrich, Oakville, ON, Canada) and zinc chloride (ZnCl_2_, 100 mM) (Sigma Aldrich) stock solutions were prepared and stored in demineralized water.

#### Preparation of Tomatidine

Tomatidine (Sigma Aldrich) was solubilized in dimethylsulfoxide (DMSO) at a concentration of 4.5 mM and stored at −20°C until use.

### Biofilm Assays

#### Microtiter Plate Assay

*Listeria monocytogenes* isolates, used for biofilm production, were grown overnight in Tryptic Soy Broth medium with 0.6% Yeast Extract (TSBYE) at 30°C as previously described (Cherifi et al., 2017).

Briefly, 100 μl of the overnight bacterial culture adjusted to an OD_600 nm_ of 1 were suspended in 10 ml of fresh BHI. Then, 100 μl of the newly inoculated BHI were distributed into wells of 96-well microplates (Corning Costar #3595, United States). Wells were covered with parafilm to avoid evaporation. After incubation at 30°C for 24 h, the microplate was washed three times. Then, the bacterial biofilms were stained with 150 μl of a solution of crystal violet at 0.1% for 20 min and rinsed with ultra-pure water until the wash-liquid was clear. The absorbed crystal violet was then eluted from attached cells with 96% ethanol (200 μl/well) and the quantification was carried out by measuring the OD_595 nm_. To investigate the dose-dependant effect of CNS supernatants on biofilm formation, wells were inoculated with *L*. *monocytogenes* strains resuspended in a BHI including several SN ratios (Table 2). EDTA, zinc chloride and tomatidine were tested according to the concentrations indicated in Table 2. Biofilm formation was then performed as described above.

**TABLE 2:** Concentrations used for antibiofilm assays and corresponding controls.

	**SN_*CNS*_ (C, D, E, F, H)**	**EDTA**	**ZnCl_2_**	**Tomatidine**
Test (final concentration)	6–50% (v/v)	100 μM	50–1,000 μM	33–220 μM
Controls	BHIG	Demineralized water	Demineralized water	DMSO

The protocol was slightly modified to investigate the impact on bacterial adhesion. After mixing cultures with active compounds, cells were allowed to attach for 4 h. After this adhesion step, we operated in two different ways. The first was a direct visualization and a quantification of attached cells, as described above. The second technique consisted of washing the wells, adding fresh medium and allowing the biofilm to mature for 24 h. Biofilms were then quantified as described above.

To study the ability to disturb or disperse mature biofilms, the following protocol was used. Biofilms were grown during 24 h in microplates, wells were then washed, and active molecules were added at the appropriate concentrations. The microtiter plate was incubated for an additional 24 h at 30°C and biofilms were quantified using crystal violet as described above.

#### Biofilm Formation Using a Microfluidic System

Dynamic biofilm formation was followed using the BioFlux 200 system with 48-well plates (Fluxion biosciences, South San Francisco, CA, United States). The protocol was adapted for *L. monocytogenes* biofilm by Cherifi et al. (2017). Briefly, cells of an overnight bacterial culture were centrifuged 20 min at 4,000 × *g* and resuspended in fresh pre-warmed BHI medium for controls or mixed with SN, EDTA, zinc chloride and tomatidine at the same concentrations described above for the static biofilm assays (Table 2). One hundred microliters of these cultures were added to the output wells of the BioFlux plate and injected at 0.05 Pa of shear stress for 30 s into the microfluidic channels. Cells were then allowed to attach during 4 h at 30°C without flow.

After the adhesion step, the input wells were filled with 1.25 ml of pre-warmed BHI diluted with demineralized water at 1/10 (v/v) and the 48-well plate was incubated at 30°C with a constant flow of 50 μl/h.

Microscopic observations were performed by confocal laser scanning microscopy (CLSM; Olympus FV1000 IX81). The biofilms formed were observed by staining cells with FilmTracerTM FM 1-43^®^ fluorescent marker (Molecular Probes; Eugene, OR, United States) as recommended by the manufacturer. The biofilm stacks were then analyzed with Image-Pro software (version 9.0; Media Cybernetics, Inc., United States) to estimate the thicknesses (μm) and the biovolume (μm^3^.μm^–2^) of biofilms.

### Antibacterial Assays

Target bacteria grown overnight were resuspended in BHI and mixed with the antimicrobial compound solutions or SN at the appropriate concentrations. Cultures were incubated 24 h at 30°C. Growth was monitored by measuring culture turbidity at 600 nm at various time points.

### Autoaggregation Assay

The effect of active compounds on the autoaggregation of *Listeria* strains was evaluated using the method described by Gowrishankar et al. (2016). Five ml of BHI, with or without active compounds, were inoculated with overnight cell suspensions (10^7^ cfu/ml) and incubated at 30°C for 24 h. Then, 0.5 ml was aspirated from the top of the cultures and optical density were measured at 600 nm (OD 1). The rest of the cultures were vortexed and optical density were measured at 600 nm (OD 2). The degree of autoaggregation was calculated as follows:

%autoaggregation=((OD 2-OD 1)/OD 2)×100

### Cell Motility Assay

Swimming assays were performed as follows. TSBYE agar plates (0.3% of agar) were prepared by supplementing the active molecules at the selected concentrations in TSBYE. An overnight culture of *L. monocytogenes* was used for centric spot inoculation of agar plates and incubation was conducted in an upright position at 30°C for 24 h). After incubation, the diameter of the swimming motility migration was compared to the negative control.

### Statistical Analyses

Each experiment was performed in independent triplicates. Statistical analyses were done using GraphPad Prism software (Version 5.03). Comparisons were carried out either by the Student *t*-test (significant if *p*-values were < 0.05) or by using one-way analysis of variance (ANOVA) followed by Tukey’s multiple comparison test (set at 5%).

## Results

### CNS Supernatants, Zinc Chloride, EDTA, and Tomatine Inhibit Biofilm Formation of *Listeria monocytogenes* Under Static Conditions

**FIGURE 1:**
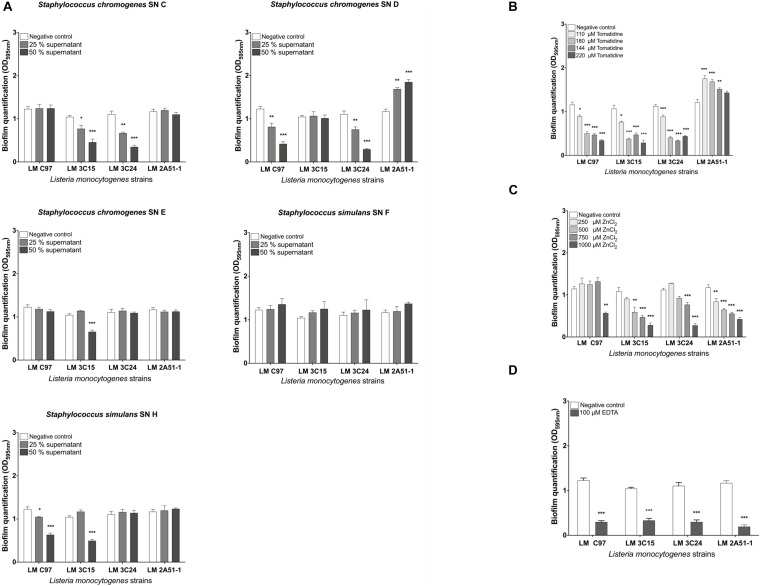
Screening of antibiofilm activities against *Listeria monocytogenes* strains in microtiter plate. **(A)** CNS supernatant antibiofilm activity. **(B)** Tomatidine antibiofilm activity. **(C)** Zinc chloride antibiofilm activity. **(D)** EDTA antibiofilm activity. Data were analyzed by ANOVA test. *p < 0.05; **p < 0.01; ***p < 0.001.

This first step consists in screening the antibiofilm activities of all the selected molecules. All observed antibiofilm effects were dose-dependent (Figure 1). The antibiofilm activity of CNS supernatants (SN C, D, E, F, and H) was assessed *in vitro* by quantifying crystal violet bound to biofilm-embedded bacterial cells. Results are shown in Figure 1A. *Staphylococcus chromogenes* supernatants C and D significantly (*p* < 0.05) inhibited biofilm formation of *L. monocytogenes* 3C15 and 3C24, and C97 and 3C24, respectively, in a dose-dependant manner (>50% of inhibition at a concentration of 50%, Figure 1A). *S. chromogenes* supernatant E and *S. simulans* supernatant H significantly (*p* < 0.05) inhibited biofilm formation of *L. monocytogenes* 3C15, and C97 and 3C15, respectively (>50% of inhibition at a concentration of 50%, Figure 1A). Tomatidine also in a dose-dependant manner significantly (*p* < 0.05) inhibited biofilm formation of 3C15, C97, and 3C24 (≥50% of inhibition at a concentration ≥144 μM, Figure 1B). Noteworthy, formation of biofilm by strain 2A51-1 was insensitive to tomatidine and to all CNS supernatants used in this study. Interestingly, an increase of biofilm production was observed for strain 2A51-1 in presence of SN D or tomatidine (>30%) but tomatidine was still able to reduce this burst of biofilm production to the initial level (control culture without test molecules) at the highest concentration tested (220 μM). Zinc chloride, used at 1,000 μM, showed a significant antibiofilm activity against all *Listeria* strains (Figure 1C). In fact, it was just as effective as EDTA, the positive control (>60% of inhibition, Figure 1D).

In light of these findings, only the molecules which exhibited antibiofilm activity were retained for the rest of the study.

### Visualization of Antibiofilm Activities Against *Listeria monocytogenes* Grown in a Microfluidic System

**FIGURE 2:**
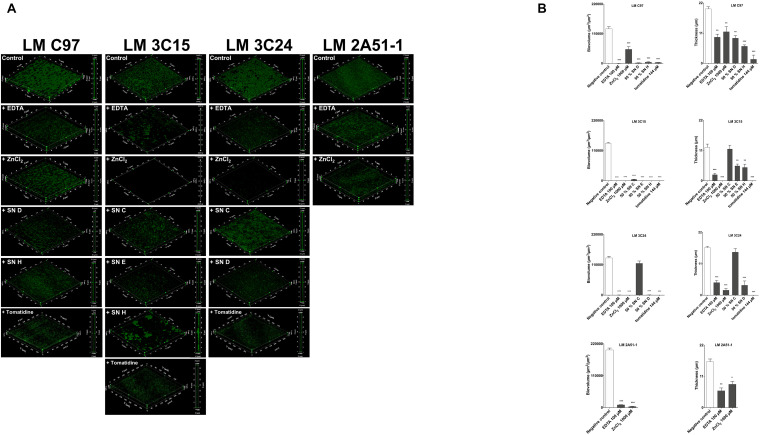
Antibiofilm activity against *Listeria monocytogenes* strains in a microfluidic system. **(A)** 3D representation and a side view projection. **(B)** Average thickness and biovolumes calculated, for each experiment, from Image Pro analyses of 10 images stacks obtained from two independent biofilms. Data were analyzed by ANOVA test. *p < 0.05; **p < 0.01; ***p < 0.001.

The effects of the screened molecules on biofilms were investigated using a BioFlux system to confirm effectiveness under dynamic conditions. Reductions of biofilms were observed with exposure to compounds (Figure 2A). Further, stacks analysis, using the Image-Pro software, showed a significant reduction in the thickness and biovolume of biofilms (Figure 2B).

As observed in microtiter plates, EDTA (at 100 μM) treatment was highly effective against all *L. monocytogenes* strains tested (Figure 2A). Biofilm thickness and biovolumes were greatly reduced (>50 and >80%, respectively; Figure 2A). ZnCl_2_ (at 1,000 μM) was also very effective and especially against LM 3C15 and 3C24 (Figures 2A,B). Almost all the CNS supernatants selected (at a concentration of 50%) confirmed their antibiofilm activities against LM C97, LM 3C15, and LM 3C24 (Figure 2A). Biofilm quantification values confirmed visual observations (Figure 2B). In fact, high biovolumes and thickness decreases were obtained (>50%). An exception was observed concerning the thickness of LM 3C15 and LM 3C24 biofilms treated with SN C. Indeed, biovolumes were significantly reduced without any effect on the thickness average and this can be explained by non-uniform distribution of the biofilm on the surface. Tomatidine antibiofilm activity (at 144 μM) was also confirmed under these dynamic conditions. *L. monocytogenes* C97, 3C15, and 3C24 biofilms were considerably affected by the presence of tomatidine (>80% on biovolume and thickness, Figures 2A,B).

These findings clearly demonstrated that antibiofilm molecules screened in microtiter plates were also effective under the presence of shear force in a microfluidic system.

### *Listeria monocytogenes* Growth Inhibition

**FIGURE 3:**
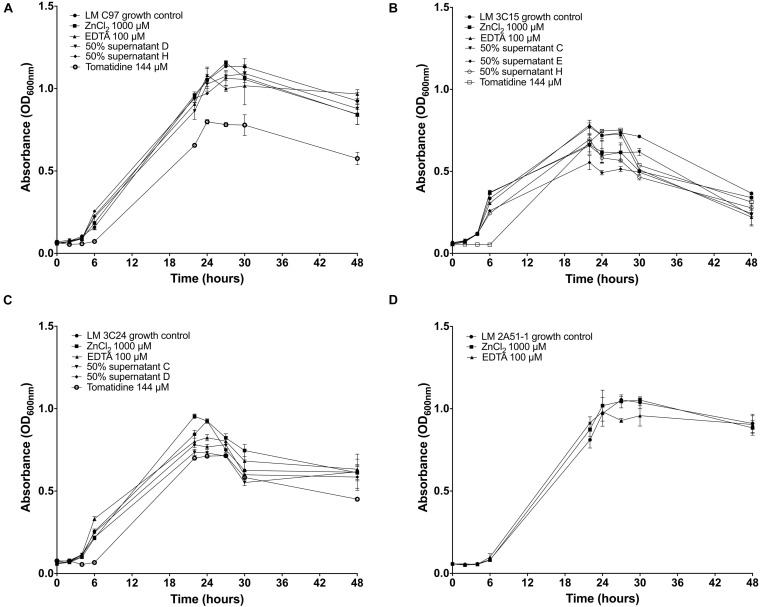
Growth curves of *Listeria monocytogenes* strains incubated or not with antibiofilm compounds. **(A)** Growth of LM C97. **(B)** Growth of LM 3C15. **(C)** Growth of LM 3C24. **(D)** Growth of LM 2A51-1.

We then examined if any growth inhibition activity by the test molecules could be responsible for the observed reduction of *L. monocytogenes* biofilms. Assays in broth cultures suggest that, overall, growth of all strains was not drastically affected by the SN, EDTA, and ZnCl_2_ molecules (Figure 3). The agar well diffusion assay confirmed the non-effectiveness of these treatments on growth (data not shown). The exception was with tomatidine that caused a delay of growth during the first 7 h for *L. monocytogenes* strains LM C97, LM 3C15, and LM 3C24 although the exponential phase resumed practically normally after this period of latency (Figures 3A–C).

### Antibiofilm Molecules Mainly Affect Bacterial Adhesion

To further analyze the antibiofilm activity, we examined the effect of these molecules on the onset of bacterial adhesion as well as on mature biofilms.

**FIGURE 4:**
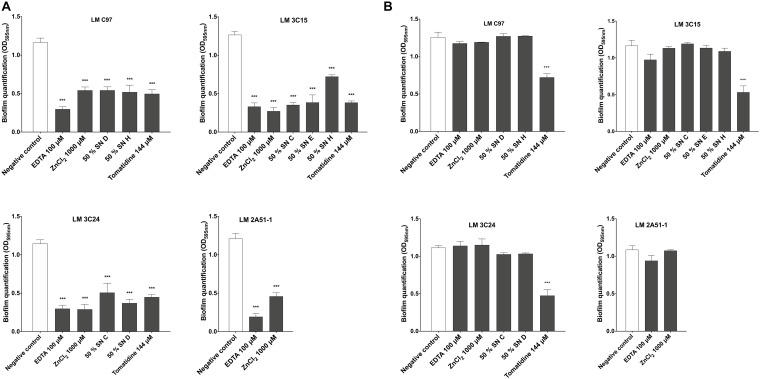
Effect of active compounds on bacterial adhesion and biofilm dispersion of *Listeria monocytogenes* strains. **(A)** Biofilm quantification after 24 h of incubation with treatments added during the adhesion step. **(B)** Compounds effects on dispersion of pre-established biofilms. Data were analyzed by ANOVA test. ***p < 0.001.

Biofilm quantification of treated cells during the 4 h adhesion step, clearly demonstrated that the effect occurred during the first hours of contact between bacterial cells and active compounds and was observable on the mature biofilm (Figure 4A). Indeed, the activity of almost all the tested molecules was expressed during the biofilm formation process. In fact, the 4 h treatment during the first stage of biofilm formation did not affect qualitatively bacterial adhesion, except for Tomatidine which provoked a significant reduction of bacterial adhesion (Supplementary Figure 1). However, an apparent modification of the cellular organization on the surface was denoted. In fact, only when treated with EDTA and ZnCl_2_, LM 2A51-1 cells were more scattered compared to the control, where the formation of microcolonies was clearly observed (Supplementary Figure 2).

The effect on dispersion has also been studied by treating preestablished biofilms. Our results (Figure 4B) revealed that investigated molecules did not induce any disruption or dispersion of pre-formed biofilms except with tomatidine against LM C97, LM 3C15, and LM 3C24.

### Antibiofilm Molecules Affect Bacterial Autoaggregation

**FIGURE 5:**
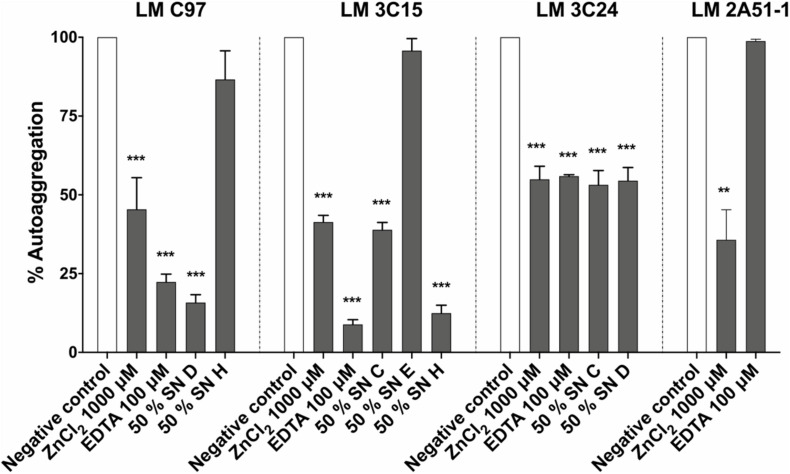
Effect of antibiofilm compounds on *Listeria monocytogenes* autoaggregation. Data were analyzed by ANOVA test. **p < 0.01; ***p < 0.001.

Bacterial autoaggregation was evaluated in order to determine the effect of antibiofilm molecules on cell-cell interaction. Results demonstrated a significant delay in autoaggregation for all the treatments, except SN H against LM-C97 and SN E against LM-3C15 (Figure 5). In all the cases, tomatidine did not induce any reduction in *L. monocytogenes* autoaggregation.

### Antibiofilm Molecules Inhibit Cell Motility

To elucidate the antibiofilm activity, *L. monocytogenes* cell motility was evaluated in order to determine whether or not there is a link between bacterial motility and biofilm formation.

**FIGURE 6:**
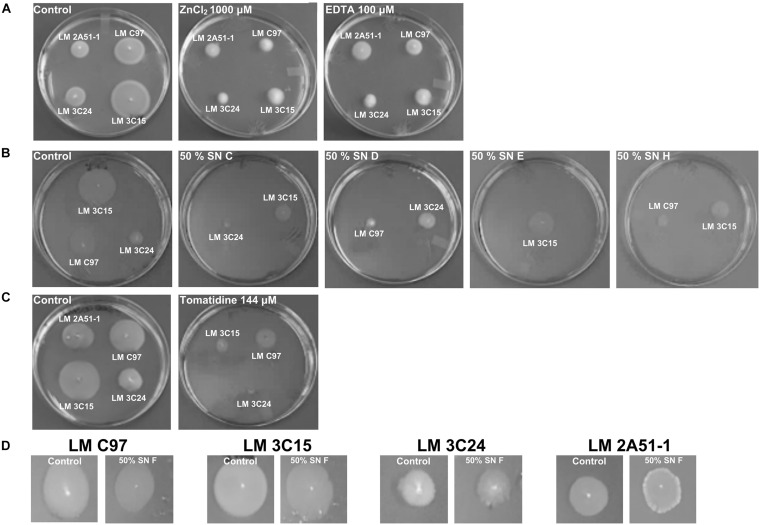
Effect of antibiofilm compounds on motility of *Listeria monocytogenes.* **(A)** Effect of ZnCl_2_ and EDTA. **(B)** Effect of CNS supernatants. **(C)** Effect of tomatidine. **(D)** Negative control: effect of SN F on motility of *Listeria monocytogenes* strains.

Molecules that showed antibiofilm activity produced visible reductions in flagella-directed swimming motility of all *L. monocytogenes* strains (Figures 6A–C). On the other hand, SN F that was devoid of antibiofilm activity (Figure 1A) did not induce any motility inhibition and served as a negative control for this assay (Figure 6D). Strain LM 2A51-1 motility, when treated with EDTA, seemed to be the only observed exception. In fact, compared to the negative control, no visual difference was observed.

## Discussion

Preventing the establishment of bacterial biofilms and the dispersion of mature biofilms are promising strategies for controlling the persistence of pathogens in food-processing industries (Yang et al., 2012).

Consequently, in this study we investigated the efficacy of compounds from different origins to affect biofilm formation of *L. monocytogenes* strains isolated from pork slaughterhouses after sanitation procedures. Compounds selection was partly based on their previously demonstrated ability to inhibit biofilm formation of other bacterial species (Chang et al., 2012; Wu et al., 2013; Goetz et al., 2017; Guay et al., 2018). The first step of this study consisted in the screening of the active molecules against *L. monocytogenes* (Figure 1). We, therefore, characterized antibiofilm activities in microtiter plate assays (static conditions) as well as in microfluidic chambers (dynamic conditions, Figure 2). *L. monocytogenes* strains showed different sensitivities to CNS culture supernatant supplementation in a dose-dependant manner. These results suggest that CNS produce exoproducts with antibiofilm activity against *L. monocytogenes*. These results are in agreement with Goetz et al. (2017) in which the authors highlighted the inhibitory effect of these CNS strains on biofilm formation of staphylococci isolates associated with bovine mastitis. Zinc chloride and EDTA exerted interesting antibiofilm activities against of all *L. monocytogenes* strains tested. Effectiveness of zinc ions was dose-dependant and 1,000 μM presented the strongest antibiofilm activity. This dose is relatively high compared to the study of Wu et al. (2013) in which authors described the effectiveness of lower concentrations of zinc (0–250 μM) against different swine pathogens. Finally, tomatidine was also, in a dose-dependent manner, effective to prevent biofilm formation of the tested strains with the exception of *L. monocytogenes* strain 2A51-1. A concentration of 144 μM was most effective against biofilm formation. These results complement work by Guay et al. (2018) which reported that although tomatidine had no antibiotic activity on its own against prototypical *Bacillales* (*i.e*., *S. aureus*, *Bacillus subtilis*, and *L. monocytogenes*), it was a very effective growth inhibitor against their respiratory deficient small colony variants (SCVs). Notably, *S. aureus* SCVs produce large amounts of biofilm and are often associated with chronic infections (Proctor, 2019).

To explain our results, different hypotheses have been considered according to biofilm development key steps. We first examined whether these compounds could display a growth inhibitory activity when added to bacterial cultures (Figure 3). Interestingly, except for tomatidine that delayed the exponential phase of growth, our experiments highlighted the lack of such antibacterial activity for CNS supernatants, zinc chloride and EDTA against free-living *L. monocytogenes* cells. These results are in agreement with other studies (Chang et al., 2012; Wu et al., 2013; Goetz et al., 2017). Up until now, few natural molecules were reported to display antibiofilm activity while being devoid of an antibacterial action. In this category, the most frequently described molecules are quorum sensing inhibitors (Estrela et al., 2009). Interestingly, the anti-virulence properties of tomatidine on *S. aureus* cells are aligned with an interference in the functions of the quorum-sensing Agr system in that species (Mitchell et al., 2012) although its principal target is energy production (Lamontagne Boulet et al., 2018), and as such this also explains the slight delay of growth caused by this molecule. Besides, most of the bacterial molecules described as anti *L. monocytogenes* are bacteriocins which are especially isolated from lactic bacteria. Crude extract of bacteriocins produced by *Lactococcus lactis* subsp. *lactis biovar* diacetylactis BGBU1-4 exerted an inhibitory effect on biofilm formation by inhibiting growth of *L. monocytogenes* (Cirkovic et al., 2016). *Lactobacillus sakei* CRL1862 also produces bacteriocins able to control *L. monocytogenes* biofilm formation (Perez-Ibarreche et al., 2016). A natural peptide derived from rice bran, named Alpep7, inhibited both growth and biofilm formation of *L. monocytogenes* (Pu and Tang, 2017). A recent study reported the antibiofilm activity of luteolin, a naturally occurring compound found in a various of plants that belongs to a group of substances called bioflavonoids. Luteolin exerts its potent antibacterial effect by impairing bacterial cell membranes and inducing cell morphological alterations in the planktonic state, as well as antibiofilm activities, by inhibiting biofilm formation and killing biofilm cells (Qian et al., 2020).

We also sought to explore the effect of our selected antibiofilm compounds on bacterial adhesion and biofilm dispersion (Figure 4). Therefore, we investigated the effects of these compounds during the first 4 h of biofilm development which corresponds to the bacterial adhesion step and on mature biofilms. On the one hand, results suggested that almost all compounds did not compromise bacterial adhesion directly. This observation allowed us to rule out the hypothesis of a physico-chemical action on the abiotic surface. The effect on the biotic surface has also been studied (Figure 5). In fact, the process of cell–cell interaction called autoaggregation depends on surface hydrophobicity of the cell (Basson et al., 2008). Thus, the observed autoaggregation reduction might be related to poor cell-cell attachment capacity and the disruption of microcolony formation. This could partially explain the antibiofilm efficacy of the active molecules. On the other hand, no dispersion of pre-established biofilms along with a destructive activity of the mature biofilm was observed. The only exception consists in the effect of tomatidine on LM C97 and this can be explained by the extended lag phase provoked by this compound.

Bacterial motility has been described as an important feature for attachment of cells to abiotic surfaces and the initiation of microcolonies on the surface (Lemon et al., 2007). For bacteria such as *Escherichia coli*, *Pseudomonas aeruginosa*, and *Vibrio cholerae*, flagella induce cell motility and might act as surface adhesins (O’Toole and Kolter, 1998; Pratt and Kolter, 1998; Watnick et al., 2001). *L. monocytogenes* is a motile bacteria which possesses four to six peritrichous flagella per cell (Schirm et al., 2004) and its flagellum-mediated motility seems critical for biofilm formation on abiotic surfaces (Lemon et al., 2007).

Our results have clearly demonstrated an inhibitory effect of all tested compounds on *Listeria* swimming motility (Figure 6). On the one hand, the role of flagella as surface adhesins has been described as minimal or dependent upon motility (Lemon et al., 2007). This may explain the absence of any inhibitory effect by our investigated compounds on the bacterial adhesion phase. However, other authors have established a causal relationship between primary adhesion and bacterial mobility. In fact, it was demonstrated that the cyclic dipeptide cyclo (L-leucyl-L-prolyl), isolated from the marine bacteria *Bacillus amyloliquefaciens*, affected preliminary steps of biofilm formation when used at a non-antibactericidal dose (Gowrishankar et al., 2016). It is therefore obvious that other factors are important to consider in trying to explain antibiofilm activities such as cell-to-cell communication, chemotaxis mechanism and the surfactant effect of the compounds. In several studies, the key role of *L. monocytogenes* flagellum in surface-associated biofilm was described as a generator of the necessary energy to overcome repulsive forces generated by the abiotic surface (Schirm et al., 2004; Lemon et al., 2007). This is in agreement with our microscopy results, since we denoted the absence/decrease of microcolonies on the abiotic surface when exposed to our selection of compounds as compared to that seen in our untreated controls (Supplementary Figure 2). It seems that even if the bacteria preserve their flagella (Supplementary Figure 3), their ability to move on the surface is hampered (data not shown).

To our knowledge, very few studies reported a relation between antibiofilm activity and bacterial motility against *L. monocytogenes*. In addition to the cyclic dipeptide cyclo(L-leucyl-L-prolyl) described above and that mitigates biofilm formation, virulence, and motility of *L. monocytogenes* (Gowrishankar et al., 2016). Sivaranjani et al. (2016) studied the antibiofilm activity of morin, a plant-derived flavonol compound, and also established the relationship between biofilm formation and motility. Recently, a new study reported the antibiofilm activity of synthetic indole. In fact, it has been shown that this molecule significantly diminished biofilm formation and related virulence of *L. monocytogenes* including motility, cell aggregation and exopolysaccharide production (Rattanaphan et al., 2020).

Results obtained in this study, together with anti-listerial recent findings, allow to consider that flagellum-mediated motility represents a promising molecular target in the fight against the persistence of *L. monocytogenes* in industrial food settings.

## Data Availability Statement

The raw data supporting the conclusions of this article will be made available by the authors, without undue reservation.

## Author Contributions

ID, TC, and CG contributed to the acquisition of data and analysis. ID drafted the manuscript. All authors contributed to the study concept, interpretation of data, revising the article, and final approval of the article.

## Conflict of Interest

The authors declare that the research was conducted in the absence of any commercial or financial relationships that could be construed as a potential conflict of interest.

## References

[B1] Allerberger F. Wagner M. (2010). Listeriosis: a resurgent foodborne infection. *Clin. Microbiol. Infect.* 16 16–23. 10.1111/j.1469-0691.2009.03109.x 20002687

[B2] Basson A. Flemming L. A. Chenia H. Y. (2008). Evaluation of adherence, hydrophobicity, aggregation, and biofilm development of *Flavobacterium johnsoniae*-like isolates. *Microb. Ecol.* 55 1–14. 10.1007/s00248-007-9245-y 17401596

[B3] Chang Y. Gu W. McLandsborough L. (2012). Low concentration of ethylenediaminetetraacetic acid (EDTA) affects biofilm formation of *Listeria monocytogenes* by inhibiting its initial adherence. *Food Microbiol.* 29 10–17. 10.1016/j.fm.2011.07.009 22029913

[B4] Chaturongakul S. Raengpradub S. Wiedmann M. Boor K. J. (2008). Modulation of stress and virulence in *Listeria monocytogenes*. *Trends Microbiol.* 16 388–396. 10.1016/j.tim.2008.05.006 18619843 PMC3400534

[B5] Cherifi T. Arsenault J. Pagotto F. Quessy S. Côté J.-C. Neira K. (2020). Distribution, diversity and persistence of *Listeria monocytogenes* in swine slaughterhouses and their association with food and human listeriosis strains. *PLoS One* 15:e0236807. 10.1371/journal.pone.0236807 32760141 PMC7410256

[B6] Cherifi T. Jacques M. Quessy S. Fravalo P. (2017). Impact of nutrient restriction on the structure of *Listeria monocytogenes* biofilm grown in a microfluidic system. *Front. Microbiol.* 8:864. 10.3389/fmicb.2017.00864 28567031 PMC5434154

[B7] Cirkovic I. Bozic D. D. Draganic V. Lozo J. Beric T. Kojic M. (2016). Licheniocin 50.2 and bacteriocins from *Lactococcus lactis* subsp. lactis biovar. diacetylactis BGBU1-4 Inhibit biofilms of coagulase negative staphylococci and *Listeria monocytogenes* clinical isolates. *PLoS One* 11:e0167995. 10.1371/journal.pone.0167995 27930711 PMC5145223

[B8] Dufour S. Labrie J. Jacques M. (2019). The Mastitis Pathogens Culture Collection. *Microbiol. Resour. Announc.* 8 e00133–e00119. 10.1128/MRA.00133-19 30975807 PMC6460030

[B9] Estrela A. B. Heck M. G. Abraham W. R. (2009). Novel approaches to control biofilm infections. *Curr. Med. Chem.* 16 1512–1530. 10.2174/092986709787909640 19355904

[B10] Evans E. W. Redmond E. C. (2014). Behavioral risk factors associated with listeriosis in the home: a review of consumer food safety studies. *J. Food Prot.* 77 510–521. 10.4315/0362-028X.JFP-13-238 24674447

[B11] Goetz C. Tremblay Y. D. N. Lamarche D. Blondeau A. Gaudreau A. M. Labrie J. (2017). Coagulase-negative staphylococci species affect biofilm formation of other coagulase-negative and coagulase-positive staphylococci. *J. Dairy Sci.* 100 6454–6464. 10.3168/jds.2017-12629 28624271

[B12] Gowrishankar S. Sivaranjani M. Kamaladevi A. Ravi A. V. Balamurugan K. Karutha Pandian S. (2016). Cyclic dipeptide cyclo(l-leucyl-l-prolyl) from marine *Bacillus amyloliquefaciens* mitigates biofilm formation and virulence in *Listeria monocytogenes*. *Pathog. Dis.* 74:ftw017. 10.1093/femspd/ftw017 26945590

[B13] Gu H. Fan D. Gao J. Zou W. Peng Z. Zhao Z. (2012). Effect of ZnCl2 on plaque growth and biofilm vitality. *Arch. Oral Biol.* 57 369–375. 10.1016/j.archoralbio.2011.10.001 22071420

[B14] Guay I. Boulanger S. Isabelle C. Brouillette E. Chagnon F. Bouarab K. (2018). Tomatidine and analog FC04-100 possess bactericidal activities against *Listeria*, *Bacillus* and *Staphylococcus* spp. *BMC Pharmacol. Toxicol.* 19:7. 10.1186/s40360-018-0197-2 29439722 PMC5812199

[B15] Hamon M. Bierne H. Cossart P. (2006). *Listeria monocytogenes*: a multifaceted model. *Nat. Rev. Microbiol.* 4 423–434. 10.1038/nrmicro1413 16710323

[B16] Kleta S. Hammerl J. A. Dieckmann R. Malorny B. Borowiak M. Halbedel S. (2017). Molecular tracing to find source of protracted invasive listeriosis outbreak, southern Germany, 2012-2016. *Emerg. Infect. Dis.* 23 1680–1683. 10.3201/eid2310.161623 28930013 PMC5621528

[B17] Lamontagne Boulet M. Isabelle C. Guay I. Brouillette E. Langlois J. P. Jacques P. E. (2018). Tomatidine is a lead antibiotic molecule that targets *Staphylococcus aureus* ATP synthase subunit C. *Antimicrob. Agents Chemother.* 62:e02197-17. 10.1128/AAC.02197-17 29610201 PMC5971568

[B18] Lemon K. P. Higgins D. E. Kolter R. (2007). Flagellar motility is critical for *Listeria monocytogenes* biofilm formation. *J. Bacteriol.* 189 4418–4424. 10.1128/JB.01967-06 17416647 PMC1913361

[B19] Mitchell G. Fugere A. Pepin Gaudreau K. Brouillette E. Frost E. H. Cantin A. M. (2013). SigB is a dominant regulator of virulence in *Staphylococcus aureus* small-colony variants. *PLoS One* 8:e65018. 10.1371/journal.pone.0065018 23705029 PMC3660380

[B20] Mitchell G. Gattuso M. Grondin G. Marsault E. Bouarab K. Malouin F. (2011). Tomatidine inhibits replication of *Staphylococcus aureus* small-colony variants in cystic fibrosis airway epithelial cells. *Antimicrob. Agents Chemother.* 55 1937–1945. 10.1128/AAC.01468-10 21357296 PMC3088192

[B21] Mitchell G. Lafrance M. Boulanger S. Seguin D. L. Guay I. Gattuso M. (2012). Tomatidine acts in synergy with aminoglycoside antibiotics against multiresistant *Staphylococcus aureus* and prevents virulence gene expression. *J. Antimicrob. Chemother.* 67 559–568. 10.1093/jac/dkr510 22129590

[B22] O’Neil H. S. Marquis H. (2006). *Listeria monocytogenes* flagella are used for motility, not as adhesins, to increase host cell invasion. *Infect. Immun.* 74 6675–6681. 10.1128/IAI.00886-06 16982842 PMC1698079

[B23] O’Toole G. A. Kolter R. (1998). Flagellar and twitching motility are necessary for *Pseudomonas aeruginosa* biofilm development. *Mol. Microbiol.* 30 295–304. 10.1046/j.1365-2958.1998.01062.x 9791175

[B24] Perez-Ibarreche M. Castellano P. Leclercq A. Vignolo G. (2016). Control of *Listeria monocytogenes* biofilms on industrial surfaces by the bacteriocin-producing *Lactobacillus sakei* CRL1862. *FEMS Microbiol. Lett.* 363:fnw118. 10.1093/femsle/fnw118 27190146

[B25] Pratt L. A. Kolter R. (1998). Genetic analysis of *Escherichia coli* biofilm formation: roles of flagella, motility, chemotaxis and type I pili. *Mol. Microbiol.* 30 285–293. 10.1046/j.1365-2958.1998.01061.x 9791174

[B26] Proctor R. (2019). Respiration and small colony variants of *Staphylococcus aureus*. *Microbiol. Spectr.* 7 1–15. 10.1128/microbiolspec.GPP3-0069-2019 31198131 PMC11257146

[B27] Pu C. Tang W. (2017). The antibacterial and antibiofilm efficacies of a liposomal peptide originating from rice bran protein against *Listeria monocytogenes*. *Food Funct.* 8 4159–4169. 10.1039/c7fo00994a 29022979

[B28] Qian W. Liu M. Fu Y. Zhang J. Liu W. Li J. Li X. (2020). Antimicrobial mechanism of luteolin against Staphylococcus aureus and Listeria monocytogenes and its antibiofilm properties. *Microb. Pathog.* 142:104056. 10.1016/j.micpath.2020.104056 32058023

[B29] Rattanaphan P. Mittraparp–Arthorn P. Srinoun K. Vuddhakul V. Tansila N. (2020). Indole signaling decreases biofilm formation and related virulence of Listeria monocytogenes. *FEMS Microbiol. Lett.* 367:fnaa116. 10.1093/femsle/fnaa116. 32658271

[B30] Reis-Teixeira F. B. D. Alves V. F. de Martinis E. C. P. (2017). Growth, viability and architecture of biofilms of *Listeria monocytogenes* formed on abiotic surfaces. *Braz. J. Microbiol.* 48 587–591. 10.1016/j.bjm.2017.01.004 28237677 PMC5498454

[B31] Schirm M. Kalmokoff M. Aubry A. Thibault P. Sandoz M. Logan S. M. (2004). Flagellin from *Listeria monocytogenes* is glycosylated with beta-O-linked N-acetylglucosamine. *J. Bacteriol.* 186 6721–6727. 10.1128/JB.186.20.6721-6727.2004 15466023 PMC522210

[B32] Sivaranjani M. Gowrishankar S. Kamaladevi A. Pandian S. K. Balamurugan K. Ravi A. V. (2016). Morin inhibits biofilm production and reduces the virulence of *Listeria monocytogenes*–an in vitro and in vivo approach. *Int. J. Food Microbiol.* 237 73–82. 10.1016/j.ijfoodmicro.2016.08.021 27543817

[B33] Sofos J. N. Geornaras I. (2010). Overview of current meat hygiene and safety risks and summary of recent studies on biofilms, and control of *Escherichia coli* O157:H7 in nonintact, and *Listeria monocytogenes* in ready-to-eat, meat products. *Meat Sci.* 86 2–14. 10.1016/j.meatsci.2010.04.015 20510532

[B34] Tauxe R. V. (2002). Emerging foodborne pathogens. *Int. J. Food Microbiol.* 78 31–41. 10.1016/s0168-1605(02)00232-5 12222636

[B35] Vatanyoopaisarn S. Nazli A. Dodd C. E. Rees C. E. Waites W. M. (2000). Effect of flagella on initial attachment of *Listeria monocytogenes* to stainless steel. *Appl. Environ. Microbiol.* 66 860–863. 10.1128/aem.66.2.860-863.2000 10653766 PMC91911

[B36] Vazquez-Boland J. A. Kuhn M. Berche P. Chakraborty T. Dominguez-Bernal G. Goebel W. (2001). Listeria pathogenesis and molecular virulence determinants. *Clin. Microbiol. Rev.* 14 584–640. 10.1128/CMR.14.3.584-640.2001 11432815 PMC88991

[B37] Watnick P. I. Lauriano C. M. Klose K. E. Croal L. Kolter R. (2001). The absence of a flagellum leads to altered colony morphology, biofilm development and virulence in *Vibrio cholerae* O139. *Mol. Microbiol.* 39 223–235. 10.1046/j.1365-2958.2001.02195.x 11136445 PMC2860545

[B38] Wu C. Labrie J. Tremblay Y. D. Haine D. Mourez M. Jacques M. (2013). Zinc as an agent for the prevention of biofilm formation by pathogenic bacteria. *J. Appl. Microbiol.* 115 30–40. 10.1111/jam.12197 23509865

[B39] Yang L. Liu Y. Wu H. Song Z. Hoiby N. Molin S. (2012). Combating biofilms. *FEMS Immunol. Med. Microbiol.* 65 146–157. 10.1111/j.1574-695X.2011.00858.x 22066868

[B40] Yin H. B. Boomer A. Chen C. H. Patel J. (2019). Antibiofilm efficacy of peptide 1018 against *Listeria monocytogenes* and shiga toxigenic *Escherichia coli* on equipment surfaces. *J. Food Prot.* 82 1837–1843. 10.4315/0362-028X.JFP-19-168 31599650

