# Supplementary material for: Counteracting Bacterial Motility: A Promising Strategy to Narrow Listeria monocytogenes Biofilm in Food Processing Industry
Source: Front Microbiol. 2021 Jun 2;12:673484. doi: 10.3389/fmicb.2021.673484 (PMC8206544; doi:10.3389/fmicb.2021.673484)
Supplement: Supplementary file 1 [file Data_Sheet_1.PDF]

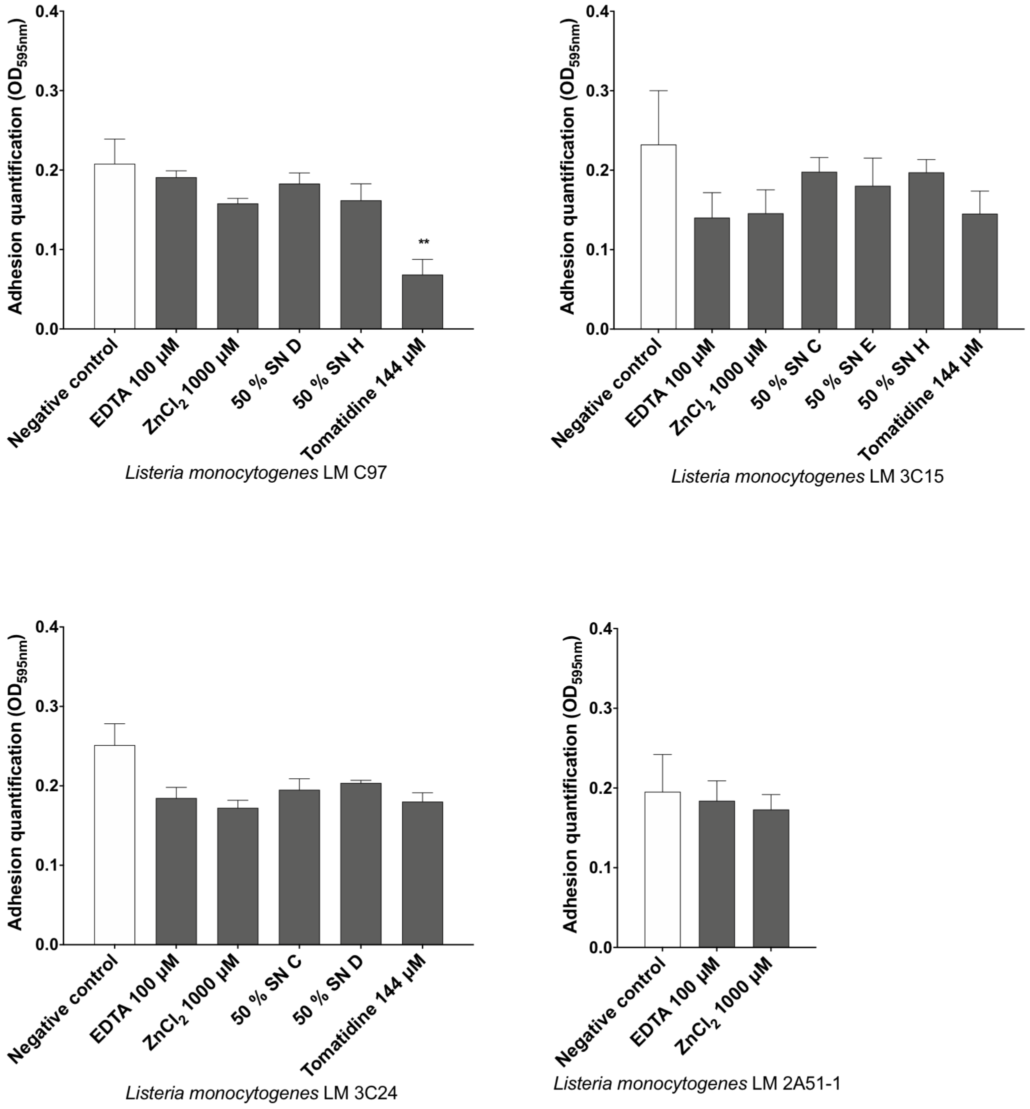

Supplementary figure 1: Bacterial quantification of *L. monocytogenes* adhered cells after a 4 h adhesion step. Data were analyzed by ANOVA test, *: $p < 0.05$; **: $p < 0.01$; ***: $p < 0.001$.
