# Supplementary material for: Counteracting Bacterial Motility: A Promising Strategy to Narrow Listeria monocytogenes Biofilm in Food Processing Industry
Source: Front Microbiol. 2021 Jun 2;12:673484. doi: 10.3389/fmicb.2021.673484 (PMC8206544; doi:10.3389/fmicb.2021.673484)
Supplement: Supplementary file 2 [file Data_Sheet_2.PDF]

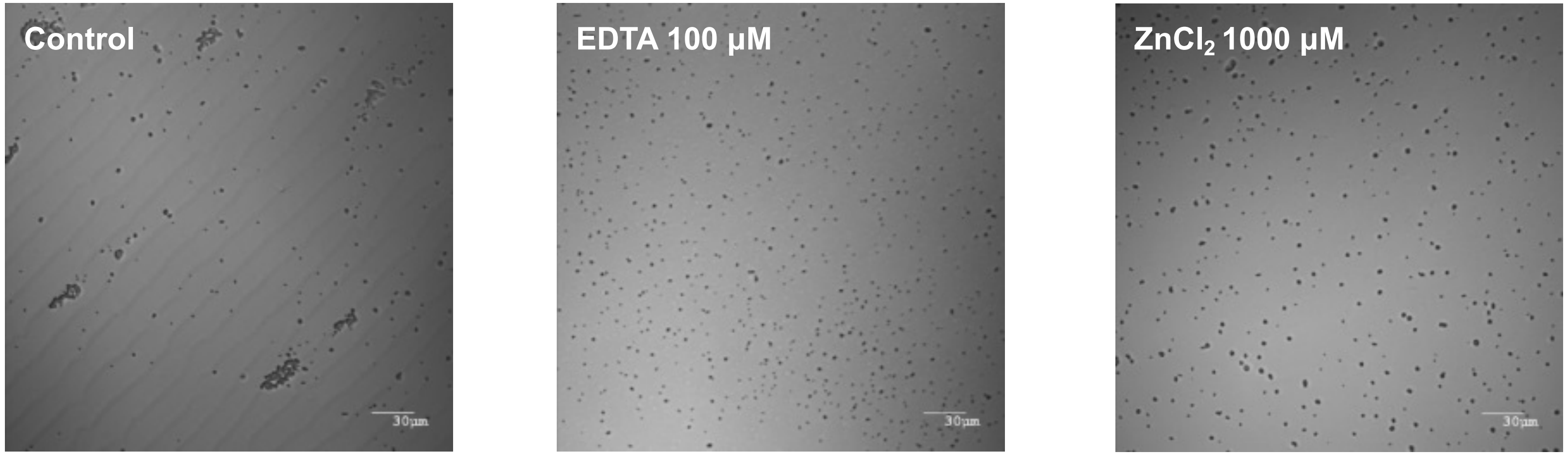

***L. monocytogenes* LM 2A51-1**

Supplementary figure 2: Microscopy visualization of bacterial adhesion of LM 2A51-1 after 4 h.
