# Supplementary material for: Counteracting Bacterial Motility: A Promising Strategy to Narrow Listeria monocytogenes Biofilm in Food Processing Industry
Source: Front Microbiol. 2021 Jun 2;12:673484. doi: 10.3389/fmicb.2021.673484 (PMC8206544; doi:10.3389/fmicb.2021.673484)
Supplement: Supplementary file 3 [file Data_Sheet_3.PDF]

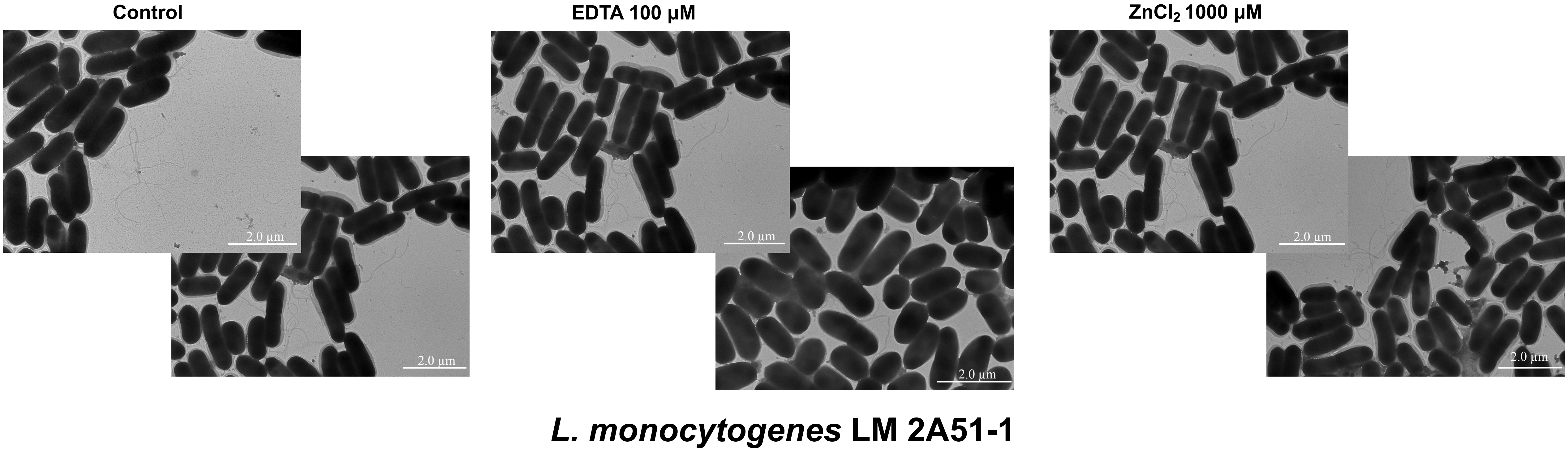

Supplementary figure 3: Transmission electron microscopic analysis of LM 2A51-1. After 24h of treatment, bacteria were fixed with a 4 % glutaraldehyde solution. Then, 100 µL of fixed bacteria (1/2 diluted in the appropriate medium) were incubated directly onto a formvar-carbon film on a 400 mesh copper grid (Electron Microscopy Sciences, Hatfield, PA, USA) overnight in a humid chamber. The grids were then negatively stained using 2 % phosphotungstic acid and visualized at 80 kV by TEM (Philips, model EM201 and Hitachi, model HT7700, Rexdale, ON, Canada).
